# LncRNA ILF3-AS1 mediated the occurrence of epilepsy through suppressing hippocampal miR-212 expression

**DOI:** 10.18632/aging.103148

**Published:** 2020-05-13

**Authors:** Xiaodong Cai, Ling Long, Chao Zeng, Guanzhong Ni, Yangyang Meng, Qiang Guo, Ziyi Chen, Zhong Li

**Affiliations:** 1 Department of Neurology, The Sixth Affiliated Hospital of Sun Yat-Sen University, Guangzhou, China; 2 Department of Neurology, The Third Affiliated Hospital of Sun Yat-Sen University, Guangzhou, China; 3 Department of Pathology, The Eight Affiliated Hospital of Sun Yat-Sen University, Shenzhen, China; 4 Department of Neurology, The First Affiliated Hospital of Sun Yat-Sen University, Guangzhou, China; 5 Department of Neurosurgery, Guangdong 999 Brain Hospital, Guangzhou, China

**Keywords:** temporal lobe epilepsy, long noncoding RNA, ILF3-AS1, miR-212, MMP

## Abstract

Increased expression of some matrix metalloproteinases (MMPs) is closely associated with epilepsy. However, factors that promote their expression have not been clarified. Long noncoding RNAs (lncRNAs) play crucial roles in the development of human diseases, including various cancers, but its potential function in temporal lobe epilepsy (TLE) has remained unexplored. In this study, we showed that hippocampal and serum ILF3-AS1 levels are higher in TLE patients than in matched controls. Interleukin (IL)-1β and tumor necrosis factor (TNF)-α induced ILF3-AS1 expression in astrocytes, while ectopic expression of ILF3-AS1 enhanced IL-6 and TNF-α expression. Ectopic ILF3-AS1 in astrocytes also increased expression of MMP2, MMP3, MMP9 and MMP14, but suppressed expression of miR-212. Consistent with that finding, miR-212 levels were lower in the hippocampus and serum of TLE patients than their controls. This suggests that ILF3-AS1 promotes expression of inflammatory cytokines and MMPs by targeting miR-212 and that ILF3-AS1 plays a crucial role in the development of TLE.

## INTRODUCTION

Epilepsy is a nervous system disorder resulting from the abnormal neuronal excitation [1–3]. Status epilepticus can cause persistent and acute damage to the nervous system and hippocampal neurons [4–6]. Hypoxia, ischemia, inflammation, and edema may occur within the hippocampus after status epilepticus [7–9]. These stimuli can induce release of excitatory amino acids from the cells, leading to Ca^2+^ and Na^+^ influx, which can in turn damage the neurons, leading to neuronal apoptosis, fiber sprouting, glial cell proliferation, loss of hippocampal neurons, and hippocampal sclerosis, among others [10–12]. Temporal lobe epilepsy (TLE) is one of the most prevalent form of the disease and often leads to hippocampal sclerosis [13, 14]. Despite some progress, the mechanism underlying TLE remains unknown.

Evidence suggests that increased expression or activity of matrix metalloproteinases (MMPs) after an insult can contribute to epileptogenesis [15–18]. It has therefore been suggested that MMP inhibition could be an effective therapy for epilepsy; however, available MMP inhibitors lack specificity and have serious side effects. The molecules upstream of MMPs may be more suitable for targeted treatment than MMPs themselves. However, the causes of the MMP upregulation in these cases is unclear.

Long noncoding RNAs (lncRNAs) are a subgroup of noncoding RNAs that are greater than 200 nucleotides in length [19–22]. Multiple studies have demonstrated that lncRNAs play key roles in the progression and pathogenesis of a variety of diseases, including cardiovascular disease, intervertebral disc degeneration, neurodegenerative diseases, and cancer [23–27]. LncRNAs were also found to be involved in numerous biological processes, including cell development, apoptosis, stem cell differentiation, migration, and inflammation [28–31]. For example, dysregulated expression of a newly identified lncRNA, ILF3-AS1, has been detected in several cancers, including colon cancer, osteosarcoma, cervical tumor, and melanoma [32–36]. In addition, two lncRNAs, H19 and RNA-UCA1, were recently shown to be involved in the development of epilepsy [37, 38]. The aim of the present study, therefore, was to investigate whether ILF3-AS1 expression is altered in TLE patients and to assess the relationships between ILF3-AS1 and MMPs. The results showed that both hippocampal and serum levels of ILF3-AS1 are higher in TLE than in the control group. Moreover, ectopic expression of ILF3-AS1 induced expression of inflammatory cytokines, MMP3 and MMP9 through targeting of miR-212.

## RESULTS

### Quantitative analysis of the subjects

In this study, 23 patients met the inclusion criteria for the TLE group and 18 patients met those for the control group. These 41 patients were included in the final analysis. Age and gender were well matched between the two groups.

### ILF3-AS1 is overexpressed in the hippocampus of TLE patients

**Figure 1:**
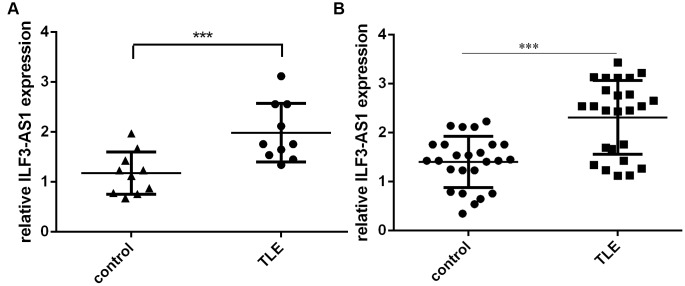
**qRT-PCR analysis showing ILF3-AS1 is upregulated in the hippocampus and serum of TLE patients.** Shown are levels of ILF3-AS1 expression in the hippocampus (**A**) and serum (**B**) of TLE patients and their matched controls. Data are shown as mean ± standard deviation (SD). ***p<0.001.

We first analyzed ILF3-AS1 expression in the hippocampus of TLE and control patients. As indicated in Figure 1A, hippocampal ILF3-AS1 expression was higher in the TLE group than in the control group. Additionally, our results showed that serum ILF3-AS1 levels were also higher in TLE than control group (Figure 1B).

### Relations between ILF3-AS1 and inflammatory cytokine expression

**Figure 2:**
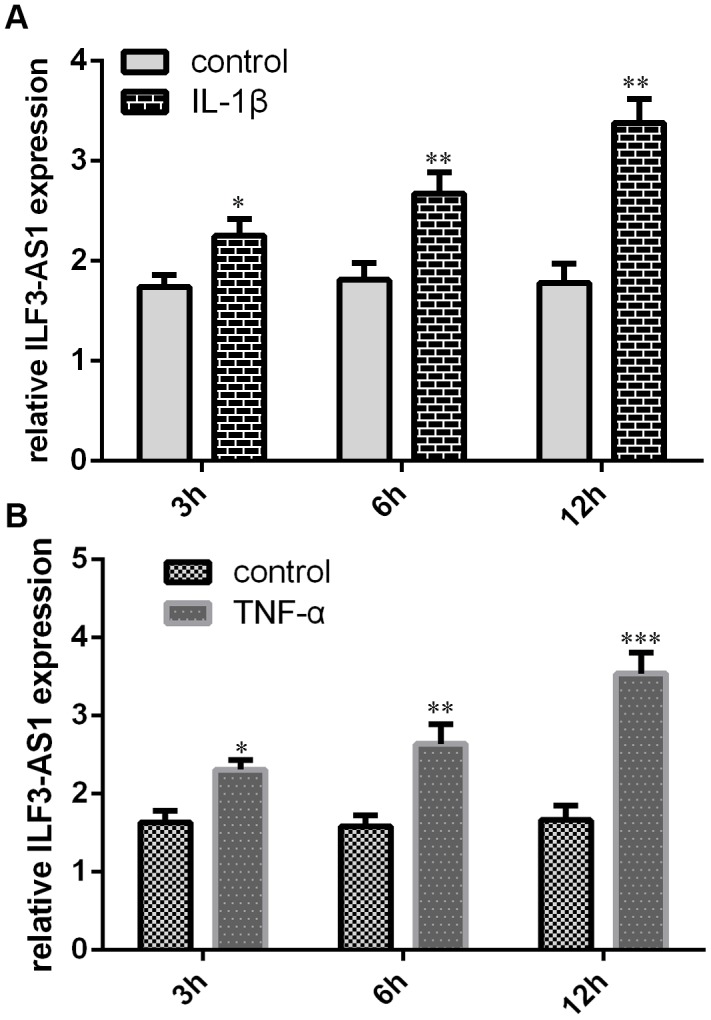
**qRT-PCR analysis showing IL-1β and TNF-α induce ILF3-AS1 expression.** Shown are levels of ILF3-AS1 expression induced by IL-1β (**A**) and TNF-α (**B**) in astrocytes. *p<0.05, **p<0.01, and ***p<0.001.

To study the effects of inflammatory cytokines on ILF3-AS1 expression in TLE, astrocytes were treated with IL-1β or TNF-α. Our data revealed that IL-1β and TNF-α were each able to induce ILF3-AS1 expression in the astrocytes (Figure 2A and 2B).

**Figure 3:**
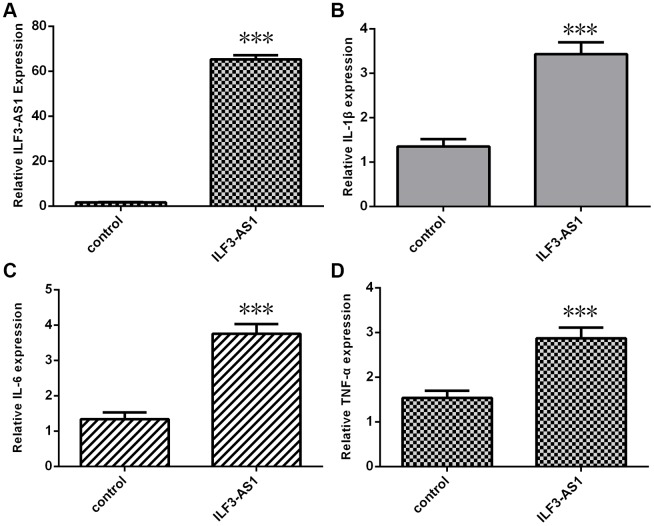
**qRT-PCR analysis showing ILF3-AS1 induces inflammatory cytokine expression.** (**A**) ILF3-AS1 expression in astrocytes transfected with pcDNA-ILF3-AS1 plasmid. Ectopic expression of ILF3-AS1 led to enhanced expression IL-1β (**B**), IL-6 (**C**), and TNF-α (**D**).***p<0.001.

To further explore the relation between ILF3-AS1 and inflammatory cytokines in TLE, astrocytes were transfected with the pcDNA-ILF3-AS1 plasmid, which led to overexpression of ILF3-AS1 in the transfectants (Figure 3A). Notably, this ectopic ILF3-AS1 expression also promoted expression of IL-1β (Figure 3B), IL-6 (Figure 3C) and TNF-α in the astrocytes (Figure 3D).

### Overexpression of ILF3-AS1 promotes MMP2, MMP3, MMP9, and MMP14 expression

**Figure 4:**
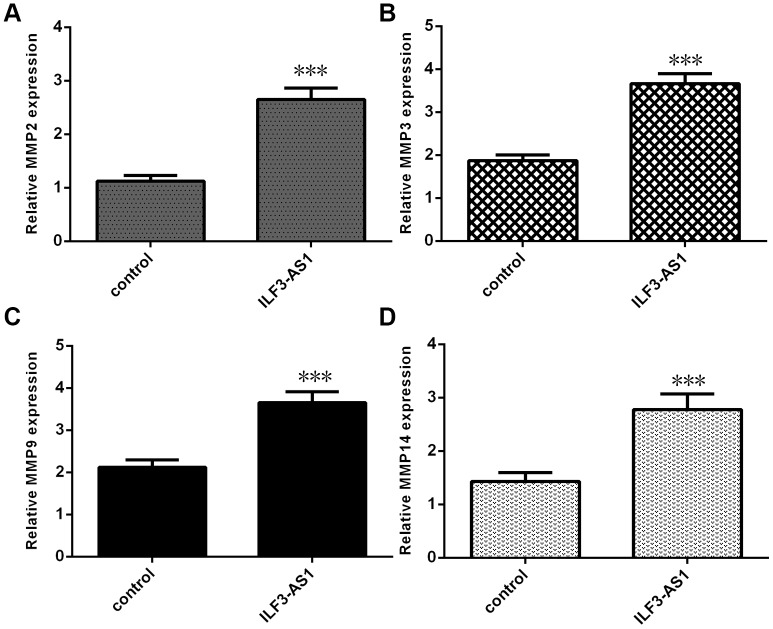
**qRT-PCR analysis showing ILF3-AS1 promotes MMP2, MMP3, MMP9, and MMP14 expression.** Ectopic expression of ILF3-AS1 in astrocytes induced expression of MMP2 (**A**), MMP3 (**B**), MMP9 (**C**), and MMP14 (**D**).***p<0.001.

Because MMP2, MMP3, MMP9, and MMP14 expression is reportedly enhanced in TLE after inflammatory cytokine stimulation [15–18], we studied whether the expression of these genes is regulated by ILF3-AS1. We found that levels of MMP2 (Figure 4A), MMP3 (Figure 4B), MMP9 (Figure 4C) and MMP14 (Figure 4D) expression were all upregulated in astrocytes ectopically overexpressing ILF3-AS1.

### ILF3-AS1 targets miR-212 in cultured astrocytes and the hippocampus of TLE patients

**Figure 5:**
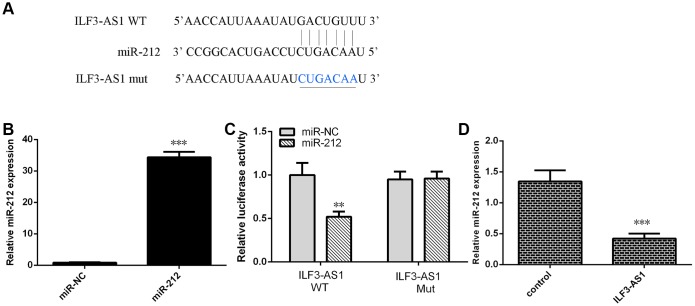
**Ectopically expressed ILF3-AS1 targets miR-212.** (**A**) Starbase (http://starbase.sysu.edu.cn/index.php) analysis predicting a binding site between ILF3-AS1 and miR-212. (**B**) qRT-PCR analysis of miR-212 expression. (**C**) ILF3-AS1-luciferase reporter assay showing miR-212 overexpression decreases ILF3-AS1 expression in astrocytes. (**D**) Forced ILF3-AS1 expression suppresses miR-212 expression in astrocytes. **p<0.01.

**Figure 6:**
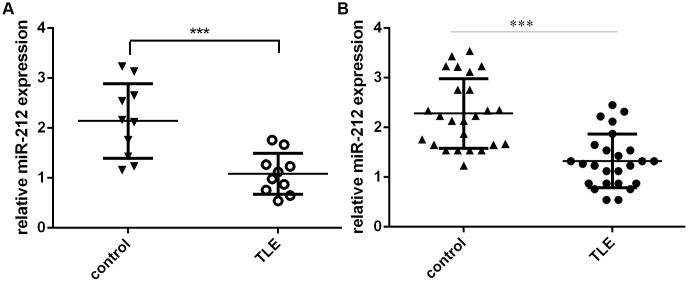
**miR-212 expression is downregulated in TLE patients.** Shown are levels of hippocampal (**A**) and serum (**B**) miR-212 expression in TLE patients and their matched controls. ***p<0.001.

To investigate the molecular mechanism by which ILF3-AS1 may contribute to TLE progression, we analyzed its interactome using Starbase (http://starbase.sysu.edu.cn/index.php), which predicted that ILF3-AS1 likely binds miR-212 (Figure 5A). We also showed that miR-212 expression was increased in astrocytes treated with miR-212 mimic (Figure 5B). Using luciferase reporter assays, we observed that miR-212 overexpression decreased luciferase activity in astrocytes transfected with a wild-type ILF3-AS1 reporter plasmid (Figure 5C). Conversely, forced ILF3-AS1 expression suppressed miR-212 expression in the cells (Figure 5D). Consistent with those findings, both hippocampal and serum miR-212 levels were lower in TLE patients than the control group (Figure 6A and 6B).

### ILF3-AS1 induces expression of inflammatory cytokines, MMP3 and MMP9 by targeting miR-212

**Figure 7:**
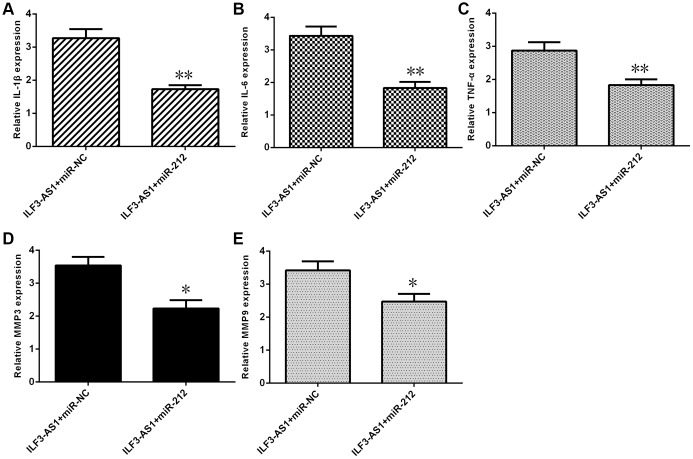
**ILF3-AS1 induces expression of inflammatory cytokines, MMP3, and MMP9 by targeting miR-212.** qRT-PCR analysis showing that forced miR-212 expression decreases expression of IL-1β (**A**), IL-6 (**B**), TNF-α (**C**), MMP3 (**D**), and MMP9 (**E**) in astrocytes ectopically expressing ILF3-AS1.*p<0.05, **p<0.01.

To determine whether ILF3-AS1 promotes expression of inflammatory cytokines and MMPs by suppressing miR-212, a series of rescue experiments were performed. Forced miR-212 expression reduced expression of IL-1β (Figure 7A), IL-6 (Figure 7B), and TNF-α (Figure 7C) in astrocytes overexpressing ILF3-AS1. Likewise, elevated miR-212 expression decreased expression of MMP3 (Figure 7D) and MMP9 (Figure 7E) in the ILF3-AS1-overexpressing cells.

## DISCUSSION

TLE is the most common form of chronic drug-resistant epilepsy, and several studies have shown that patients with chronic TLE are more likely to develop cognitive impairment and premature aging [39, 40]. Although ILF3-AS1 has been found to play roles in colon cancer, osteosarcoma, cervical cancer, and melanoma [32–36], its potential function in TLE has remained unexplored. In this paper, we found that both hippocampal and serum ILF3-AS1 levels are higher in TLE patients than matched control individuals. In addition, we also found that overexpression of ILF3-AS1 promoted expression of MMP2, MMP3, MMP9, and MMP14. Our findings are consistent with those of Rempe et al. [16], who reported that MMP2 and MMP9 activities are elevated in a TLE rat model, and with those of Korotkov et al. [18], who observed increased MMP3 expression in the hippocampus of TLE-HS patients and in the rat TLE model. Based on those results it was suggested that MMP inhibitors may be an effective for anti-epilepsy therapy [15–18]. Moreover, our findings suggest ILF3-AS1 is situated upstream of MMPs, making it a potentially useful target for epilepsy treatment.

Evidence suggests inflammation is both a cause and a consequence of TLE [41, 42]. Numerous inflammatory mediators have been identified in brain samples from patients with refractory epilepsy [43]. Korotkov et al. [18] demonstrated that miR-155 inhibition attenuates MMP3 overexpression in astrocytes after IL-1β induction, and that miR-155 and MMP3 are overexpressed in the hippocampus of TLE model and TLE patients. In the present study, we showed that the inflammatory cytokines IL-1β and TNF-α each enhanced ILF3-AS1 expression in astrocytes, and that ectopic expression of ILF3-AS1 induced expression of IL-6 and TNF-α.

Previous studies demonstrated that lncRNAs act as ceRNAs against microRNAs, serving as sponges to inhibit miRNA expression. For example, PVT1 was shown to act via the Wnt pathway to suppress astrocyte activation and induce BDNF expression in hippocampal tissues from epilepsy cases [44]. In a TLE rat model, the lncRNA H19 was found to mediate apoptosis in hippocampal neurons by downregulating let-7b expression [45]. In addition, UCA1 reportedly reduced epilepsy and seizure-caused brain injury by modulating miR-495 expression [46]. In addition, Hu et al. [36] showed that ILF3-AS1 knockdown suppressed osteosarcoma cell growth, invasion, and migration and induced apoptosis through inhibition of miR-212 expression. This prompted us to perform a luciferase reporter analysis, which showed that miR-212 overexpression leads to a decrease in ILF3-AS1-luciferase activity in astrocytes. Moreover, forced ILF3-AS1 expression suppressed miR-212 expression in the astrocytes, and hippocampal and serum miR-212 expression was lower in TLE patients than in their control group. Taken together these results suggest ILF3-AS1 targets miR-212 to induce inflammatory cytokine and MMP3 and MMP9 expression.

In summary, our findings indicate that ILF3-AS1 levels are elevated in the hippocampus and serum of TLE patients, and that ILF3-AS1 induces expression of inflammatory cytokines and MMP3 and MMP9 by targeting miR-212. This is noteworthy and suggests a therapeutic strategy targeting the ILF3-AS1/miR-212/MMP3/9 axis may be an effective approach to treating TLE.

## MATERIALS AND METHODS

### Tissues

Peripheral blood specimens were obtained from TLE patients and age- and sex-matched controls. All TLE cases met the diagnostic criteria for TLE from the International League Against Epilepsy (ILAE) [47]. Temporal cortex samples were collected from TLE cases undergoing lesionectomy; the control tissues were from patients with hypertension who needed an emergency operation to resolve an intracranial hematoma. Written consent was provided by all participants, and this research was approved by the Ethics Committee of the Sixth Affiliated Hospital of Sun Yat-Sen University.

### Cell cultures and transfections

The astrocytes were collected from the Cell Center of the Chinese Academy of Medical Sciences (Beijing, China). These cells were cultured in DMEM supplemented with 20% fetal bovine serum (FBS) and L-glutamine (Sigma, St. Louis, MO). miR-NC, miR-212 mimic, pcDNA-control, and pcDNA-ILF3-AS1 were purchased from FitGene (Guangzhou, China). A Lipofectamine 2000 kit was used to transfect astrocytes with the mimics and plasmids.

### RNA isolation and qRT-PCR

A Trizol kit was used to isolate RNA from cells and specimens. qRT-PCR assays were applied to determine the levels of ILF3-AS1, miR-212 and mRNA expression on an iQ5 qRT-PCR System (Bio-Rad, Hercules, CA) using SYBR Green (Takara Biotechnology, Dalian, China). U6 or GAPDH was utilized as the normal control. Expression level was determined using the 2^−ΔΔCt^ method. The primers utilized in this research were as follows: for ILF3-AS1, 5′-TAAACCCCACTGTCTTCC-3′ (forward) and 5′-TTCCTTGCTCTTCTTGCTC-3′ (reverse); for miR-212, 5′-TGGTGTAACAGTCTCCAGTCA-3′ (forward) and 5′-CGATGACCTATGAATTGACAGACG-3′ (reverse); and for GAPDH, 5′- CACCGTAGCCT TCCGAGTA-3′ (forward) and 5′-GCCCTTGATG AGCTGTTGA-3′ (reverse).

### Dual-luciferase reporter assays

Astrocytes were co-transfected with a reporter construct, pGL-3 control plasmid, miR-NC, or miR-212 mimic. Cells were harvested 24 h later and subjected to dual-luciferase analysis (Promega, Madison, WI). Firefly luciferase activity was normalized that of *Renilla* and expressed as the firefly/*Renilla* ratio.

### Statistical analysis

Statistical analyses were conducted using SPSS 18.0 (SPSS Inc., Chicago, IL, USA). Results are presented as the mean ± standard deviation (SD). Student’s t-test was used to evaluate the statistical significance of differences between means. Values of P<0.05 were considered significant.
